# The Relationship Between Osteoarthritis and Sarcopenia in Geriatric Diabetic Patients

**DOI:** 10.14744/SEMB.2021.42890

**Published:** 2021-12-29

**Authors:** Sema Basat, Ridvan Sivritepe, Damla Ortaboz, Ecem Sevim, Sabri Atay, Arzu Baygul

**Affiliations:** 1. Department of Internal Medicine, University of Health Sciences Turkey, Umraniye Training and Research Hospital, Istanbul, Turkey; 2. Department of Internal Medicine, Istanbul Beykoz State Hospital, Istanbul, Turkey; 3. Department of Internal Medicine, Istanbul Arnavutköy State Hospital, Istanbul, Turkey; 4. Division of Rheumatology, Hospital for Special Surgery, New York, USA; 5. Antalya Korkuteli Health Directorate, Antalya, Turkey; 6. Department of Biostatistics, Koç University Faculty of Medicine, Istanbul, Turkey

**Keywords:** Geriatrics, osteoarthritis, sarcopenia, type II diabetes mellitus

## Abstract

**Objectives:**

Osteoarthritis is a common disease affecting the quality of life in the elderly population. Osteoarthritis is a painful condition commonly encountered in patients aged 65 years and older and it may cause muscle weakness. Sarcopenia is a condition that has an increasing prevalence in the elderly population. The present study evaluated the relationship between sarcopenia and osteoarthritis.

**Methods:**

The study included 100 patients aged 65 years and older who were diagnosed with diabetes mellitus. The patients were divided into two groups as Group 1 and Group 2. Group 1 was composed of 50 patients with diabetes and osteoarthritis and Group 2 was composed of 50 patients with diabetes but without osteoarthritis. A detailed medical history was obtained from all patients and all patients underwent physical examination. The get-up and go test was performed, handgrip strength was measured with a hand dynamometer, bioimpedance analysis was performed, and mid-upper arm circumference, calf circumference and waist circumference were measured, and laboratory tests including complete blood count, biochemical nutritional parameters, liver and kidney function tests, and erythrocyte sedimentation rate were ordered. The Kellgren and Lawrence grading system was used to evaluate the severity of osteoarthritis and the skeletal muscle mass index was used to evaluate the muscle mass. These parameters were compared between the two groups.

**Results:**

Of the study participants, 1% had severe sarcopenia, 22% had moderate sarcopenia, and 77% did not have sarcopenia. Albumin (p=0.013), magnesium (p=0.038), total protein (0.004), erythrocyte sedimentation rate (p=0.047), hemoglobin level (p=0.018), muscle strength (p=0.046), height (p=0.033), and muscle mass (p<0.05) were significantly different in patients with osteoarthritis compared to patients without osteoarthritis. Patients with osteoarthritis achieved poorer results on the get-up and go test (p=0.014), and mid-upper arm circumference (p=0.028), and calf circumference (p=0.016) were lower in this group. There was a negative moderate correlation between the muscle mass and the Kellgren and Lawrence grade (p<0.05, r: −0.405), whereas there was a positive moderate correlation between sarcopenia index and the Kellgren and Lawrence grade (p<0.05, r: 0.320) in patients with osteoarthritis.

**Conclusion:**

The present study is the first to evaluate the relationship between sarcopenia and osteoarthritis in geriatric diabetic patients. The present study found a significant relationship between osteoarthritis and sarcopenia in geriatric patients with type II diabetes mellitus. The authors suggest that pain associated with osteoarthritis results in immobility, decrease in functional performance, and thus development of sarcopenia.

Diabetes mellitus is an important public health problem in Turkey as well as in the world.^[1]^ Diabetes mellitus and impaired glucose intolerance are considered to be related with aging and their prevalence rates increase with increasing age.^[2]^ There are two main polygenic defects in type II diabetes mellitus. These include insulin resistance and impairment in beta-cell insulin secretion.^[3]^ Insulin resistance plays a role in the pathophysiology of hypertension, hyperlipidemia, and obesity, and it is also thought to be involved in the development of sarcopenia.^[4,5]^ Sarcopenia, which is defined as the progressive generalized loss of muscle mass and muscle strength, is one of the most important causes of frailty, disability, and morbidity in the elderly population.^[6,7]^ Sarcopenia and associated disorders pose a significant burden on the patients and their relatives as well as on the health-care systems and economies of the countries.^[8]^ Several mechanisms are implicated in the onset and progression of sarcopenia. These mechanisms may be related to protein synthesis, proteolysis, neuromuscular integrity, and muscle fat content. Various mechanisms can be involved in sarcopenic individuals and their relative contribution may vary over time. The pathophysiology of sarcopenia related with aging can be explained by a decrease in anabolic hormones (testosterone, estrogen, growth hormone, and insulin-like growth factor-1 [IGF-1]), increase in apoptotic activity of myofibrils, increase in proinflammatory cytokines (tumor necrosis factor-alpha [TNF-α] and interleukin [IL]-6), increased oxidative stress associated with free radical accumulation, alterations in mitochondrial functions of myocytes, and decrease in the number of α-motor neurons.^[9]^ Chronic disorders as osteoarthritis are catabolic processes contributing to the progression of sarcopenia.^[10]^ Considering the fact that common factors could affect changes in the different components of the musculoskeletal system, it is possible that osteoarthritis could predict age-related sarcopenia.^[11,12]^ Osteoarthritis is the most common joint disorder occurring in patients aged 65 years and older at a rate of 40% and affecting the knee joint.^[13,14]^ It is difficult to suggest a clear relationship between osteoarthritis and diabetes; however, many studies have demonstrated a positive relationship between these two entities. Several authors have suggested that the prevalence of osteoarthritis is higher in young and middle-aged diabetic patients, and joint destruction starts at an earlier age and shows a more severe course than in the control group. It was demonstrated that insulin resistance and hyperinsulinemia stimulates bone growth in patients with type II diabetes mellitus.^[15]^

Multiple mechanisms are involved in the pathogenesis of sarcopenia and osteoarthritis both of which have an increasing prevalence among the middle-aged and elderly diabetic population and are associated with a decline in physical function and decrease in the quality of life. These two conditions also constitute an important public health concern due to treatment costs, associated complications and increasing prevalence in recent years in Turkey as well as in the world. The aim of the present study was to evaluate the relationship between sarcopenia and osteoarthritis, which is one of the musculoskeletal disorders responsible for severe disability that occurs with aging in diabetic patients.

## Methods

The study was designed as a cross-sectional study and has been approved by the local ethics committee. (The Ethics Committee of University of Health Sciences Umraniye Traning and Research University Hospital, Date: 22.01,2016; Number: 1154) The study included 100 consecutive patients aged 65 years and older who were admitted to the diabetes outpatient clinics at our hospital. The patients were divided into two groups. Group 1 was composed of diabetic patients who presented with joint pain and were diagnosed with osteoarthritis and who did not have any other conditions that would cause sarcopenia. Group 2 was composed of diabetic patients who did not have joint pain and in whom the diagnosis of osteoarthritis was ruled out. Patients with type I diabetes mellitus, patients with a history of major surgery, malignancy, severe cardiovascular disease, acute cerebrovascular conditions, acute or chronic infections, uncontrolled diabetes, major psychiatric condition, patients with abnormal kidney and liver function tests, patients with a pacemaker or any type of implant, severe edema, severe electrolyte disturbances, patients with any condition that would affect mobility (cerebrovascular accident, end-stage dementia, hip dislocation, extremity injury caused by traffic accident, etc.), and patients with diabetic neuropathy or polyneuropathy and those with additional chronic conditions that would cause sarcopenia were excluded from the study. Weight, height, body mass index, waist circumference, and blood pressure of the patients were measured. The get-up and go test was performed, handgrip strength was measured with a hand dynamometer, bioimpedance analysis (BIA) was performed, and mid-upper arm circumference, calf circumference and waist circumference were measured. Laboratory tests including complete blood count, biochemical nutritional parameters, liver and kidney function tests, and erythrocyte sedimentation rate were ordered. Fasting blood samples were collected in the morning between 08:00 AM and 10:00 AM. Blood samples were collected into SST II, LH PST II, and EDTA tubes, and simultaneously analyzed. Handgrip strength was measured using a hand dynamometer. Anthropometric measurements including height, mid-upper arm circumference, and calf circumference were obtained. The Kellgren and Lawrence grade and skeletal muscle mass index (SMMI) were calculated and these values were compared between the two groups.

### Metabolic Parameters

Plasma glucose was measured with an enzymatic test, glycated hemoglobin was measured using high-performance liquid chromatography (HPLC) method. Total cholesterol, high-density lipoprotein, low-density lipoprotein, calcium, phosphor, alanine transaminase, aspartate transaminase, gamma-glutamyl transferase, alkaline phosphatase, amylase, and albumin, and triglyceride concentrations were measured using enzymatic colorimetric test. Creatinine level was measured with Jaffe reaction, Vitamin D level was measured using HPLC method, bilirubin levels were measured using the diazo reaction. C-reactive protein level was measured using enzyme immunoassay, iron, iron binding capacity, magnesium, total protein, uric acid, and blood urea nitrogen were measured using spectrophotometry, ferritin level was measured using immunochemiluminescence, and folat level was measured using radioimmunoassay. Potassium, sodium, and chloride levels were measured by ion-selective electrode, creatine kinase and lactic dehydrogenase activity were measured by kinetic analysis method. Lipase levels were measured by turbidimetric method, erythrocyte sedimentation rate was measured by the Westergren method. TSH, free T3, free T4 and Vitamin B12 levels were measured by electrochemiluminescence, and hemogram parameters were measured by flow cytometry.

### Anthropometric Measurements

Height was measured using a stadiometer (Ekoter mechanical stadiometer with scale), while the shoes, socks, and hats were removed. Weight was measured using a daily-calibrated electronic scale (Ekoter mechanical stadiometer with scale), while the shoes, socks, and heavy garments were removed. The body mass index (BMI; kg/m^2^) was calculated using these measurements. The mid-upper arm circumference and calf circumference were measured using a tape. A mid-upper arm circumference <22 cm was considered low and a value above 22 cm was considered normal; a calf circumference <31 cm was considered low; and a value above 31 cm was considered normal.^[16]^

### Assessment of Osteoarthritis

Knee osteoarthritis was assessed by a standing semiflexed anterior-posterior radiograph as per the Altman atlas.^[17]^ Each radiograph was assessed by the common opinion of three assessors as per the Altman atlas. The Kellgren and Lawrence grading system was used to evaluate the severity of osteoarthritis.^[18]^ The patient were divided into five groups as the following: Grade 0 = Normal, Grade 1 = suspected, possible joint space narrowing and subtle osteophytes, Grade 2 = mild, definite osteophytes and possible joint space narrowing, Grade 3 = moderate, multiple moderate osteophytes, definite joint space narrowing, some sclerosis, and possible epiphyseal deformity, and Grade 4 = severe, large osteophytes, gross loss of joint space, marked sclerosis, and definite epiphyseal deformity.

### Definition of Sarcopenia

The SMMI was calculated using weight, muscle mass (%), and BIA with the following formula: SMMI (kg) = ([height^2^/resistance × 0.401] + [gender × 3.825] + [age × −0.071]) + 5.102. Height was measured in centimeters, resistance was measured in Ohm, 1 point was assigned to male gender and 0 points was assigned to female gender, and age was evaluated in years. The cutoff values for SMMI in males are as follows: ≥10.76 kg/m^2^, normal; 8.51–10.75 kg/m^2^, moderate sarcopenia: ≤8.50 kg/m^2^, and severe sarcopenia.^[19,20]^

### Handgrip Strength

The right and left handgrip strength was measured using a hydraulic hand dynamometer (JAMAR hydraulic hand dynamometer, Sammons Preston). Handgrip strength in both sides was measured three times while the patient was in seated position with the arm positioned next to the trunk and the elbow flexed at 90°, and the average of three measurements was recorded.^[21]^

### Physical Performance

The physical performance was evaluated with the get-up and go test. The patients were instructed to stand up from a chair without holding the armrests, walk 3 m and turn around, walk back to the chair, and then sit down. The performance score was evaluated as follows: 1 = normal, 2 = subtly abnormal, 3 = mildly abnormal, 4 = moderately abnormal, and 5 = severely abnormal. The score of patients showing no evidence for the risk of falls during the test was considered to be normal and the score of patients showing any evidence for the risk of falls during the test was considered to be severely abnormal.^[22]^

### Statistical Analysis

Descriptive statistics (mean, standard deviation, minimum, median, and maximum) were used to define continuous variables. The Student’s t-test was used to compare two independent variables with normal distribution, and the Mann–Whitney U test was used to compare two variables without normal distribution. Pearson’s correlation coefficient was used to evaluate the relationship between two variables with normal distribution, and Spearman’s rho correlation coefficient was calculated to evaluate the relationship between two variables without normal distribution. Chi-square test was used (Fisher’s Exact test, where appropriate) to evaluate the relationship between categorical variables. The level of statistical significance was set at 0.05. The statistical analysis was performed using MedCalc Statistical Software 12.7.7 (MedCalc Software bvba, Ostend, Belgium; http://www.medcalc.org; 2013).

## Results

Of 100 patients included in the study, 35 were male and 65 were female. The mean age was 71.3 + 5.3 years. When patients were categorized according to the sarcopenia classification system proposed by the European Working Group on Sarcopenia in Older People (EWGSOP), 1% had severe sarcopenia, 22% had moderate sarcopenia, and 77% did not have sarcopenia. Demographic data, anthropometric measurements, and clinical and biochemical parameters are summarized in Table 1.

**Table 1.** Demographic data, anthropometric measurements, and clinical parameters

	**N**	**%**
Sarcopenia		
Serious	1	1
Intermediate	22	22
No sarcopenia	77	77
Get up and go test		
Normal	38	38
Very light normal	34	34
Slightly normal	21	21
Moderately abnormal	5	5
Severely abnormal	1	1
Upper arm circumference (cm)		
Bad	12	12
Good	88	88
Calf circumference (cm)		
Bad	23	23
Good	77	77
Hand dynamometer (kg)		
Bad	55	55
Good	45	45
Osteoarthritis		
Yes	50	50
No	50	50
Kellgren lawrence		
Stage 0	48	48.5
Stage 1	18	18.2
Stage 2	19	19.2
Stage 3	10	10.1
Stage 4	4	4

There were significant differences between patients with and without osteoarthritis in terms of albumin (p=0.013), magnesium (p=0.038), total protein (p=0.004), ESR (p=0.047), hemoglobin level (p=0.018), muscle strength (p=0.046), height (p=0.033), and muscle mass (p<0.05). Patients with osteoarthritis achieved significantly worse scores in the get-up and go test (p=0.014), and mid-upper arm circumference (p=0.028) and calf circumference (p=0.016) were lower. In patients with osteoarthritis, there was a moderate negative correlation between the muscle mass and the Kellgren and Lawrence grade (p<0.01, r: −0.405), and there was a moderate positive correlation between the SMMI and the Kellgren and Lawrence grade (p<0.05, r: 0.320) (Table 2).

**Table 2.** The correlation between the SMMI/Muscle Mass and the Kellgren-Lawrence grade

**Osteoarthritis**	**Kellgren Lawrence**
Muscle mass	
Yes	−0.405*
No	0.088
SMMI*	
Yes	0.320*
No	−0.007

*SMMI: Skeletal muscle mass index.

There was significant difference between patients with moderate sarcopenia and non-sarcopenic patients in terms of amylase (p=0.013), iron (p=0.028), potassium (p=0.007), protein (p=0.043), ESR (p<0.05), hemoglobin (p<0.05), handgrip strength (p<0.05), height (p< 0.05), muscle mass (p<0.05), gender (p<0.05), the get-up and go test (p<0.05), Kellgren and Lawrence grade (p=0.020), and BMI (p<0.05) (Table 3).

**Table 3.** The different parameters between with moderate sarcopenia and non-sarcopenic

**Sarcopenia**	**Average**	**Median**	**Standard deviation**	**Minimum**	**Maximum**	**p**
Amylase (25–90 u/l)						
Middle	72.1	71.5	21.5	35	100	0.013
No	61.1	55	28.2	18	172	
Iron (50–170 ug/dL)						
Middle	77.9	70.5	31.3	21	144	0.028
No	61.7	59	22.6	16	117	
Potassium (3.5–5.4 mEq/L)						
Middle	4.4	4.4	0.5	3.6	5.7	0.007
No	4.7	4.6	0.6	2.4	5.9	
Protein (6.4–8.3 g/l)						
Middle	7.4	7.6	0.9	4.2	8.5	0.043
No	7.1	7.3	0.9	4.2	9.1	
Sedimentation (<20 mm/hour)						
Middle	17.1	9.5	14.1	4	45	<0.05
No	34.6	35	20.4	4	80	
Hemoglobin (12–15.5 g/dl)						
Middle	13.5	13.5	1.2	10.9	16.2	<0.05
No	12.3	12.3	1.5	7.5	16.2	
Hand Dynamometer (kg)						
Middle	33.1	35	11.1	12	56	<0.05
No	19.8	18	7.8	4	46	
Height (kg)						
Middle	167.5	167.5	5.05	157	177	<0.05
No	155.4	155	8.1	141	175	
Muscle mass (kg)						
Middle	56.1	53.7	5.2	40.1	64.2	<0.05
No	45.9	44.7	7.8	29.3	70.5	
BMI (kg/m^2^)*						
Middle	28	27.9	4.6	20.4	38.3	<0.05
No	32.4	32.1	5.5	18.7	49.4	

*BMI (kg/m^2^): Body mass index.

## Discussion

To the best of our knowledge, this is the first study to evaluate the association between sarcopenia and osteoarthritis in geriatric diabetic patients. The term sarcopenia (in Greek, sarx for flex and penia for loss) has been proposed to describe the loss of muscle strength associated with decreased muscle mass, decline in muscle functions and aging, and it is a complex syndrome resulting in disability and dependence with the progression of disease.^[23,24]^ Sarcopenia, which is defined as a relatively new geriatric syndrome, has become a significant burden on the healthcare systems particularly in the developed countries due to increasing prevalence in the elderly population.^[8,25,26]^ In the literature, the prevalence of sarcopenia in women aged 50 years or older was reported to be ranging between 1% and 30%.^[27]^ Sarcopenia has a complex pathophysiology in the elderly. Both intrinsic and extrinsic factors have been implicated in the development of sarcopenia. Intrinsic factors include decrease in anabolic hormones (testosterone, estrogen, growth hormone, and IGF-1), increased apoptotic activity in the myofibrils, increased levels of proinflammatory cytokines (particularly TNF-α and IL-6), oxidative stress associated with the accumulation of free radicals, changes in mitochondrial functions in the myocytes, and decrease in the number of α-motor neurons,^[27]^ whereas extrinsic factors include energy deficiency, decreased protein intake, and immobility. On the other hand, the presence of acute and chronic diseases can also contribute to the development of sarcopenia in the elderly individuals. Osteoarthritis is an important and common disease resulting in morbidity and mortality.^[28]^ There are limited data regarding the prevalence of osteoarthritis in the population.^[29]^ It has long been known that there is a relationship between chronic diseases and impaired life quality and increased risk of mortality and morbidity.^[30]^

The present study found a significant association between osteoarthritis and sarcopenia. Recent studies have shown that osteoarthritis in the knee and hip joint in the elderly people results in a decrease in the muscle mass and muscle strength.^[31,32]^ This relation can be explained by arthrogenic muscle inhibition, which is referred to as the decrease in the efferent motor neuron stimulation in the skeletal muscle, by the changes in the afferent component of the involved joint.^[33]^ Kemnitz et al. showed that knee and hip pain in patients with osteoarthritis causes a decrease in the muscle strength and quality in the lower extremities and therefore results in an increased risk of falls; they also reported that disability is not related to the degree of radiographic changes but strongly related to the severity of these changes.^[34]^ The present study showed that the presence of osteoarthritis results in a decrease in the muscle strength, mass, and performance and this was related to the degree of radiographic findings. Radiographic stage increased with increasing severity of sarcopenia and decreasing muscle mass. The study found a relationship between radiological severity and osteoarthritis. Skeletal muscle system develops as a whole. Thus, any problem in particular system may affect other systems. On the other hand, muscular system acts as an endocrine organ by producing bioactive molecules that may contribute to homeostatic regulation of both bone and muscle tissues.^[11]^ In a study conducted on German women, the prevalence of sarcopenia was found to be higher among participants with osteoarthritis in the hip joint and lower extremities. In same study, osteoarthritis was reported to cause the development of sarcopenia in elderly women.^[35]^

The present study found a negative correlation between the muscle mass measured with BIA as recommended by EWGSOP and osteoarthritis. The muscle mass was lower in patients with osteoarthritis. Different results may have been obtained if the muscle mass of the individuals were measured with more sensitive dual-energy X-ray absorptiometry. However, SMMI was not significantly different between the two groups, because calculation of SMMI does not take into account the muscle mass of the patients. This finding can be explained in this manner. Although SMMI was not different in the patient group with osteoarthritis, muscle strength and muscle performance that are other indicators of sarcopenia were lower in this group. Quadriceps muscle weakness commonly associated with knee osteoarthritis is often thought to be caused by the inactivity and impairment in the muscle caused by the pain in the involved joint. The studies have shown that quadriceps weakness can also result in osteoarthritis among other risk factors such as obesity, occupation, and gender.^[36]^ Slemenda et al. evaluated the relationship between knee osteoarthritis and lower extremity weakness and found a relationship between quadriceps muscle weakness and osteoarthritis. The same study also found quadriceps muscle weakness in patients with osteoarthritis in the absence of knee pain or muscle atrophy and this was explained by the presence of muscle function. Quadriceps muscle weakness was defined as the primary risk factor for the progression of joint destruction in patients with knee osteoarthritis.^[33]^ A similar study conducted on in-patients reported a relationship between quadriceps muscle weakness and being confined to bed and a similar relationship has been proposed for other people in the community.^[37]^

In the present study, patients with osteoarthritis had lower muscle strength. The study group in this study was composed of patients with knee osteoarthritis. Thus, we did not expect that handgrip strength measuring with hand dynamometer would be affected; however, handgrip strength showing the muscle strength was lower (19.5) in patients with osteoarthritis. This raises the possibility that there might a decrease in overall muscle strength and not only in the muscles around the involved joints. In a study reporting on males with hip osteoarthritis, muscle strength in the adductor, abductor and flexor muscles of the hip joint were found to be lower in patients with osteoarthritis.^[31]^ Another study conducted on patients with and without knee pain showed a relationship between quadriceps muscle strength and activation and knee pain.^[38]^ The get-up and go test evaluated the muscle performance, which is a component of sarcopenia.^[39]^ Quadriceps muscle plays an important role in climbing up the stairs, walking and standing, and weakness in this muscle directly causes loss of function in the patients.^[38]^ In the present study, patients with osteoarthritis achieved poorer scores in the get-up and go test compared to those without osteoarthritis. Waters et al. found significantly lower walking speed in patients with osteoarthritis than in those without osteoarthritis.^[40]^ Another study showed limitation in the activities in subjects with knee pain. Same study also showed a relationship between quadriceps muscle weakness and disability in patients with knee pain.^[38]^

The rates of patients with a mid-upper arm circumference lower than 20 cm and a calf circumference lower than 31 cm were higher among patients with osteoarthritis. These anthropometric measurements can be regarded as indirect indications of sarcopenia.

Total protein, albumin, complete blood count, and magnesium levels were lower in patients with osteoarthritis. This can be regarded as the reflection of inflammatory process related to osteoarthritis.

## Conclusion

There was a significant association between osteoarthritis and sarcopenia in geriatric patients with type 2 diabetes mellitus. The association between osteoarthritis and sarcopenia could be mediated by functional and cellular pathways. The authors consider that osteoarthritis results in sarcopenia by causing immobility and a decrease in functional performance. Clinical implication of these findings would be that physicians should be aware of increased risk of sarcopenia in patients with osteoarthritis. Detection of sarcopenia in such patients is particular importance for developing therapeutic and preventive strategies.

It is currently difficult to answer the causality dilemma of which came first, osteoarthritis, or sarcopenia?

### Disclosures

**Ethics Committee Approval:** The Ethics Committee of University of Health Sciences Umraniye Education and Research Hospital, Date: 22.01,2016; Number: 1154.

**Peer-review:** Externally peer-reviewed.

**Conflict of Interest:** None declared.

**Authorship Contributions:** Concept – S.B, R.S.; Design –S.B, R.S.; Supervision – S.B.; Materials –R.S., D.O., E.S.; Data collection &/or processing – R.S., D.O., E.S., A.B; Analysis and/or interpretation – S.B., A.B.; Literature search – R.S., D.O., E.S., S.A.; Writing – R.S., S.A.; Critical review –S.B.
